# Supplementary material for: Techniques for the surgical correction of lagophthalmos secondary to leprosy: A systematic review
Source: PLoS Negl Trop Dis. 2025 Jun 16;19(6):e0013200. doi: 10.1371/journal.pntd.0013200 (PMC12193040; doi:10.1371/journal.pntd.0013200)
Supplement: S1 File — (DOCX) [file pntd.0013200.s002.docx]

Appendix 2 – Search strands

PubMed (16/11/2024)

| Number | Search | Results |
| --- | --- | --- |
| #1 | "leprosy"[MeSH Terms] OR "hansen’s disease"[Title/Abstract] OR "hansens disease"[Title/Abstract] OR "Leprosy"[Title/Abstract] | 27,962 |
| #2 | Surgery [MESH Terms] OR "Surger*"[Title/Abstract] OR "Correct*"[Title/Abstract] OR "Treat*"[Title/Abstract] | 10,750,458 |
| #3 | Lagophthalmos [MESH Terms] OR "Lagophthalmos"[Title/Abstract] | 1,189 |
| #4 | #1 AND #2 AND #3 | 66 |

Web of Science Core Collection (16/11/2024)

| Number | Search | Results |
| --- | --- | --- |
| #1 | (TS=(leprosy)) OR TS=(Hansen’s disease) OR TS=(Hansens disease) | 17,709 |
| #2 | (TS=(Surg*) OR TS=(correct *)) OR TS=(treat*) | 9,945,420 |
| #3 | TS=(Lagophthalmos) | 1,029 |
| #4 | #1 AND #2 AND #3 | 48 |

Infolep (16/11/2024)

| Number | Search | Results |
| --- | --- | --- |
| #1 | Leprosy OR Hansen’s Disease OR Hansens Disease | 30,226 |
| #2 | Lagophthalmos | 133 |
| #3 | (Surg* OR Correc* OR treat*) | 8,157 |
| #4 | #1 AND #2 AND #3 | 87 |

#

Medline ALL (16/11/2024)

| Number | Search | Results |
| --- | --- | --- |
| #1 | Hansen's Disease.mp. or Leprosy/ or Leprosy.mp. OR Hansens Disease.mp. | 27,958 |
| #2 | (Surg* or Treat* or Correct*).mp. | 10,478,152 |
| #3 | Lagophthalmos/ or Lagophthalmos.mp. | 1,189 |
| #4 | #1 AND #2 AND #3 | 71 |
