# Supplementary material for: Techniques for the surgical correction of lagophthalmos secondary to leprosy: A systematic review
Source: PLoS Negl Trop Dis. 2025 Jun 16;19(6):e0013200. doi: 10.1371/journal.pntd.0013200 (PMC12193040; doi:10.1371/journal.pntd.0013200)
Supplement: S2 File — (DOCX) [file pntd.0013200.s003.docx]

| **Author and Title** | **Study Methodology/ Design** | **Country/ Region and setting** | **Population/ Sample** | **Surgical Technique** | **Outcomes post-surgery** | **Length of Follow-up** | **Reported Adverse Effects** | **Other Relevant Data** |
| --- | --- | --- | --- | --- | --- | --- | --- | --- |
| Surgical treatment of lagophthalmos in leprosy by the Gillies temporalis transfer  J G ANDERSEN  [1] | Prospective study – 1-3 months follow up post procedure | Karigi, Tamil Nadu, India,  Schieffelin Leprosy Research Sanatorium, Karigiri | Operated on ten cases of lagophthalmos  between 3^rd^ August 1960 and 2^nd^ November 1960 | Andersen’s modification of Gilles Temporalis Transfer | Full active closure on slight effort: eight out of ten cases. Eye closed during sleep: eight out of ten cases.  Lacrymal duct patent post-operatively: nine out of nine cases. The one case where the duct was closed pre-operatively maintained the closed duct.  Exposure symptoms arrested at the end of four weeks: nine out of nine cases. (The two eyes that did not obtain full closure on effort and had the cornea covered during sleep, have obtained full relief from exposure symptoms.) | one to three months follow-up | post-operative ~oedema | Paper states Tarsorrhaphy - quick and simple method that can be performed by relatively unskilled doctors. Is effective but leaves unsightly result. Since a more reliable method is available it should be considered. Circumocular sling is more complicated. Is effective but does not give complete closure of the eye on effort or during sleep. Has no advantage over temporalis transfer.  Transfer of a sector of the temporalis muscle to activate a circumocular sling of its own attached fascia is the most satisfactory method. Fascia is in natural continuity with muscle. No sutures are needed to fix fascia to muscle. |
| Efficacy of temporalis muscle transfer for correction of lagophthalmos in leprosy  Premal Das, Julius Kumar, G Karthikeyan, P S S Rao  [2] | Retrospective analysis of 69 cases treated by TMT during the period 1998–2009. | Uttar Pradesh, India.  Leprosy referral centre | 69 patients,101 eyes. 61 male, 8 female. Average age was 38.6. | Temporalis Muscle Transfer (TMT)  Johnsons technique | 85% of the eyes could achieve full lid closure with no measurable gap. Mean (SD) lid gap on forced closure was 4·8 (2·8) mm pre-operatively and 0·2 (0·5) mm at the end of the in-patient stay. The mean (SD) lid gap on gentle closure was 7·9 (2·6) mm preoperatively and 2·4 (1·8) mm post-operatively. The mean (SD) vertical inter-palpebral distance, during straight gaze, was reduced from 12·6 (1·6) pre-operatively to 9·8 (1·2) post- operatively. Exposure keratitis cleared in 16 of 27 eyes (60%) and Epiphora cleared or improved in 31 eyes.  In the three patients with an established opacity, it persisted post-operatively. | 5 years | Exposure keratitis and epiphora | Concluded that the TMT is a successful option (cosmetically and functionally) for correction of lagophthalmos.  Temporalis tendon transfer by this modification of Johnson’s method is a relatively easy procedure. The procedure uses a small bundle of the temporalis tendon and though invasive, does not require sophisticated equipment or magnification, so can be performed at a peripheral hospital in surgical camps by a trained surgeon. Thereby access for the community is improved. It has simple modifications which give excellent results in terms of closure of the lid gap, protection of the cornea, reversal of corneal inflammatory changes, re-establishing tear flow and eliminates the facial asymmetry typically produced by lagophthalmos. The modifications prevent the late occurrence of an ‘epicanthal fold’,5 stitch abscesses (zero), unsightly muscle bulges or tenting fascial slips. |
| Dynamic temporalis muscle transfer revisited –  a Technique for correction of lagophthalmos in  leprosy patients  Manish Singhal, Rupnarayan Bhattacharya, Shilpi Jain  [3] | Prospective study, 20 lagophthalmos patients who were operated with a minimum follow-up period of 6 months | India | 20 lagophthalmos patients. 12(60%) were male and 8(40%) were female. Median age of male cases was 35 yrs and that of female cases was 42.5 yrs. Overall median age was 36 yrs.  Mean duration of lagophthalmos is 2.54 yrs in male and 4.25 yrs in females. Mean duration in all cases was 3.22 yrs. Eleven male and seven female cases had unilateral lagophthalmos. Bilateral lagophthalmos was found in a single male and female each. | Dynamic temporalis myofascial sling transfer.  Anderson’s modification of Gillies procedure was done in 13 cases  Johnson’s operation was done in 7 cases. | Mean lid gap (in mm) during straight gaze at pre op time was 12mm, 9.8 mm at post op time and 9.1mm at 3mth and 9.0mm at 6mth follow up period. Mean lid gap in gentle closure was found to be 7.8 mm at pre op time, 2.54 mm at post op  time at 3 wks,1.9mm at 3 mth and 1.59mm at 6 mth follow up period. Mean lid gap in forced closure of eyes was 4.13mm in pre op period, 0.9 mm at 3 wks post op and 0.5 mm in both 3 and 6 mth post op follow up period.  16 (72%) patients reported excellent results with complete eyelid closure with chewing. Good result with eyelid closure (within 1-2mm) with chewing was found in 4(18%) cases and fair result with incomplete eyelid closure with chewing and compromised corneal protection was found in single (4.54%) case. | Minimum follow-up period of 6 months. |  | The temporalis muscle transfer ensured a good functional eyelid closure thus preventing corneal exposure and progression to impairment of visual acuity. It gave a satisfactory aesthetic appearance to the eye. |
| Modified silicone sling assisted temporalis muscle transfer in the management of lagophthalmos  Gupta, Ramesh C; Kushwaha, Raj NS; Budhiraja, Ina; Gupta, Priyanka; Singh, Parul  [4] | Prospective interventional study. | Uttar Pradesh, India | 10 patients including eight males and two females. Mean age of surgery was 43.9 years. | Modified temporalis muscle transfer (TMT) by silicone sling. | The procedure was highly successful in 80% (eight) patients (lid gap <1 mm). The mean (standard deviation (SD)) lid gap on eye closure was 7.7 (0.86) mm preoperatively, 0.5 (0.47) mm at 1^st^ postoperative day, and 0.7 (0.75) mm at 3^rd^ month. There was a reduction in mean lid gap on eye closure of 7 mm at 3 months (*P* < 0.0001) which is highly significant. The mean (SD) vertical interpalpebral distance during primary gaze was 12.05 (1.12) mm preoperatively, 10 (0.94) mm at 1^st^postoperative day, and 10.35 (1.08) mm at 3^rd^ month. There was a reduction in mean vertical inter palpebral distance of 1.7 mm at 3 months (*P* = 0.001) which is significant. Exposure keratitis decreased in five out of six patients at 3 months. | 3 months | Undercorrection was noted in two patients after the surgery. The gap remained the same (at 1^st^ day and 3^rd^ month) in one of the two patients (1.5 mm), but since there was no exposure, nothing was done. The gap increased in one patient from 1 mm at day 1 of surgery to 2.5 mm at 3^rd^ month of follow-up; and since there was corneal exposure, retightening of the sling was required. | The problem faced with the procedures using fascia lata was attributed to difficulty in its harvesting.  Lid loading with gold implants is simpler to perform, but results in disruption of the tear film and irritation of the conjunctiva due to delayed closure of eyelids.[^9^] Similarly; migration, extrusion, and ptosis have also been reported after gold implantation.[^10^] It can also produce foreign body reactions. On the other hand, TMT restores active movement to paralyzed eyelids with high rates of success. Furthermore, no nerve or muscle is sacrificed, scar is hidden in hair bearing area. Several publications have reported the outcome of this procedure but have recommended further improvements in the operative procedure.  Classic Gillies turnover technique has significant disadvantages like unesthetic hollowing of temporal fossa, an obvious bulge over zygomatic arch, loss of excursion of muscle due to turnover, and extensive difficult dissection; thus requiring high surgical skills.  McLauglin's technique needed intraoral dissection. Undue tension of fascial fixation may lead to unesthetic asymmetrical eyelid fissure.  the additional advantage with our procedure is that no fascia lata sling dissection has to be carried out. Silicone slings are commonly available and also economical to use. Our surgical procedure is easier to perform than previous techniques and the surgical time is greatly reduced with significantly reduced morbidity to the patient. Moreover, there were no significant postoperative complications with our procedure. |
| An evaluation of Gillies' procedure for lagophthalmos in leprosy  N Verma, S P Garg, V K Kalra, G Fromberg  [5] | Retrospective analysis of cases | Delhi, India,  Leprosy Home at Shahdara, Delhi | Twenty cases of unilateral and bilateral lagophthalmos with varying degree of exposure keratitis were chosen from among 1016 inmates.  Their ages varied from 21 to 52 years. Fifteen were males. The duration of lagophthalmos varied from 1 to 4 years. All patients had one or more lateral tarsorraphies done that had failed. | Temporalis transfer pro­cedure (modified Gillies method) |  |  | Formation of a cold abscess at the muscle fascia junction (in a case with pulmonary tuberculosis).  Delayed post-operative haemorrhage (two patients). | It has been our experience that this is the most efficient, logical physiological and cosmetically acceptable solution to the problem of lagophthalmos.  Gillies' procedure is preferable to Johnson's procedure (which essentially dif­fers in, that fascia lata is used) because a single site is operated upon.  The bump formed by the turned over temporalis muscle gets incorporated within the hair line and is not a cosmetic blemish. Since there are no lid fissure anomalies, complete correction of ectropion and cessation of epiphora, many patients (including women) now prefer this procedure to the conventional tarsorraphy. The muscle is constantly reinforced while eat­ing and so is lid closure. The tension of the sling gives enough coverage to the globe so that the cornea is covered even in those individuals who sleep with their mouth open.  We recommend this procedure for all patients physically fit to withstand it, those with exposure keratitis and lower lid paralytic ectropion. |
| Temporalis Muscle Transfer for the Treatment of Lagophthalmos in Patients with Leprosy  Refinement in Surgical Techniques to Prevent Postoperative Ptosis  Ahn, Sung yul MD; Park, Hyang Joon MD; Kim, Jong Pill MD; Park, Tae Hwan MD  [6] | Retrospective study from January 2011 to December 2014. | Hansen Welfare Association medical clinic, Incheon Korea from January 2011 to October 2014. | Seventy-five TMT operations in 60 patients were performed between 2011 and 2014. The mean age was 70.1.  All patients were diagnosed with lagophthalmos and completely healed from leprosy.  26 men and 34 women.  15 had bilateral TMT performed | 4 different temporalis muscle transfer (TMT) methods - Brown-McDowell (12)  McCord-Codner (33)  modified Gillies-Anderson (8) and modified Gillies (7)  All performed under local anaesthesia with intravenous sedation. | Brown-McDowell showed 2 to 3mm lid gap McCord-Codner showed 2 mm or less. | Not stated | 14 cases (18.7%) of postoperative ptosis.  Disadvantage of McCord- Codner is excessive traction vector to lateral side of fascia entering lateral canthal angle. Can reduce lid gap to 2 mm or less, but experienced postoperative ptosis in 21.4% (7 of 33) of the patients.  Postoperative ptosis varied based on technique. Brown-McDowell and McCord-Codner methods showed 21.4%, whilst there was no postoperative ptosis with modified Gillies method.  Gillies method resulted in a 15.4% incidence of postoperative ptosis. | McCord-Codner adopted a short muscle flap different from conventional Gillies-Anderson method, leading to less postoperative nerve injury and short operation time.  Although free muscle transfer is not appropriate in some conditions and it requires high microsurgical techniques and multifactorial considerations, the most effective method of smile restoration by far is the association of cross-facial nerve grafting and free-muscle transplantation  25% of patients who were grafted with 1.0 to 1.4 g gold plate underwent removal of the implant because of the feeling of heaviness or spontaneous discomfort. We think that weight of the implant grafted in the upper eyelid is affected with eyelid closure function.  In the treatment of lagophthalmos, the purpose of TMT is not to achieve full lid closure, but functional and aesthetic one. A total of 2 mm lid gap does not affect corneal dryness by protective effect of Bell's phenomenon. |
| The Lateral Tarsal Strip for Paralytic Ectropion in Patients with Leprosy  Mihn-Sook Jue, Jisook Yoo, Min-Soo Kim , Hyang-Joon Park  [7] | Retrospective study. | Korean Hansen Welfare Association Hospital between January 2010 and December 2015. | 40 Korean patients (44 eyelids because of bilateral lesions in 4 patients.). 26 males and 14 females, were between the ages of 53 and 87 years (mean 71.4±7.9 years). All of them were cured leprosy patients and did not respond to conservative treatments. Eyelids of most patients showed moderate degree of ectropion (n=35); 5 eyelids showed mild degree, 4 eyelids showed marked degree, and none had extreme degree of ectropion. | Lateral canthoplasty / Lateral tarsal strip procedure | Most patients were satisfied with the results and mean satisfaction scale was 2.6/3. | Average follow-up period was 12 months. | Recurrence was observed in 5 cases (5/44, 11.4%). Of 5, 2 eyelids were marked degree of ectropion (2/3, 66.7%) and 3 had moderate degree of ectropion (3/32, 9.4%).  Were no serious postoperative complications except mild size discrepancy of about 1~2 mm in both eyes in 2 patients. | The most common surgical technique for repair of ectropion is LTS, which is a quick and easy procedure.  Whereas the LTS procedure is suited for mild and moderate degree of ectropion, patients with marked and extreme degree need additional or more radical methods such as wedge resection in the former and temporalis muscle transfer in the latter[^8^](https://pmc.ncbi.nlm.nih.gov/articles/PMC5705356/#B8). Recurrence was seen in 5 eyelids and the recurrence rate was 11.4% (5/44). While the patients with mild and moderate degree of ectropion showed recurrence of 0% (0/5) and 9.4% (3/32) respectively, 66.7% (2/3) of the patients with marked degree of ectropion recurred during follow-up period. Thus, the LTS is much more suitable for mild to moderate degree of paralytic ectropion, and other or additional procedure is necessary for the patients with marked degree of ectropion. Since the patients showing recurrence in moderate degree of ectropion were in late eighth and ninth decades, senile change may be related to recurrence. |
| Temporalis muscle transfer in the correction of lagophthalmos due to leprosy  D Soares, M Chew  [8] | Retrospective and prospective study, operations were performed between 1963 and 1995 (29 since 1992). Data was collected prospectively on patients operated after 1st January 1994. Follow-up data for patients operated prior to 1 994 was obtained when they presented for review for eye or other problems | Kathmandu, Nepal,  Anandaban Leprosy Hospital | 51 TMT operations in 35 patients (27 male and 8 female) between 1963 and 1995. (29 since 1992). Average age was 46·3 years (range 18-78 years). Twenty-eight operations were on the left eye (55%), and 23 were on the right (45%). The average duration of lagophthalmos prior to operation was 8·0 years.  Sixteen patients had bilateral TMT’s; 3 operations were redone due to failure. | Temporalis muscle transfer (TMT). Johnson procedure was used in 47 eyes and the Gillies method was used in 4 eyes. | Average lid gap preoperatively on light closure was 7.3 mm which was reduced to 3.2 mm on final follow-up. The average lid gap pre-operatively on tight closure was 5.3 mm which was reduced to 0.4 mm at final follow-up.  At discharge 16 out of 27 patients had no lid gap on tight closure with another 5 patients having a lid gap of 1 mm and 6 patients having a lid gap of 3 mm or more. At final follow-up 23 out of 30 patients had no lid gap on tight closure with another 4  patients having a lid gap of 1 mm and only 1 patient having a lid gap of 3 mm.  60% of eyes at discharge and 77% of eyes at final follow-up achieved full closure.  In all patients there was a reduction in lid gap on light and tight closure. | Average duration between operation and follow-up was 7 years 3 months (range 101 days-22 years 7 months). The average duration between operation and discharge was 60 days. | Ectropion in 6 eyes (12%) -in 4 of these (4/17) the operation was performed before 1990. Since 1991 the technique of creating a suture sling to ensure that the slip remained in the lid margin has been used and the complication has only been seen in 2 out of 33 eyes.  Ptosis in 3 eyes (6%). | Correction of lagophthalmos by tarsorrhaphy and/or wedge excision of part of the lid is unsatisfactory. It is cosmetically unacceptable to many patients and often leads to increased irritation of the cornea by the scar on the lid margin. It also fails to provide closure of the eye.  Temporalis muscle transfer provides a good cosmetic result and in the person with corneal sensation provides the effector limb of the reflex arc.  Temporalis muscle transfer also serves to reduce the irritation caused to a dry cornea by lagophthalmos. In patients with normal sensation the average blink frequency preoperatively was 20 per minute possibly reflecting corneal irritation. At discharge the blink frequency was 10 per minute. At final follow-up it was 14 per minute.  In patients with severe lagophthalmos (a lid gap on tight closure of more than 5 rom) the TMT reduces the lid gap while maintaining lid mobility so that on light closure as in  Sleep the eye is protected.  Some surgeons use TMT only for those patients with sensitive corneas and use tarsorrhaphy for those with anaesthetic corneas. However, for the patients with severe lagophthalmos tarsorrhaphy fails to protect the cornea. Even if the patient does a strong blink using the TMT 3 times a minute (average blink frequency at follow-up) then the whole cornea is moistened, whereas with a tarsorrhaphy the inferior aspect of the cornea remains exposed leading to keratitis. It is very difficult to achieve lid closure and protection of the eye using only medial and/or lateral tarsorrhaphy in patients with a lid gap on tight closure exceeding 5 mm. There is a need for long-term follow-up of vision in patients with severe lagophthalmos who have had either TMT or tarsorrhaphy. However, in our experience in patients with severe lagophthalmos TMT is the operation of choice. |
| Modified tarsorrhaphy versus gold weight implant technique for paralytic lagophthalmos treatment in patients with leprosy: One-year observation of a randomized controlled trial study  Yunia Irawati, Michelle Eva Rebeca Natalia, Tjahjono D Gondhowiardjo, Ishandono Dachlan, Hardyanto Soebono  [9] | Multicenter, Prospective Randomized Open-label Blinded-Endpoint (PROBE) clinical trial, parallel-group study conducted in Indonesia (three sites). | Indonesia (three hospitals) | 23 eyes, with 11 eyes in the MT group (intervention group) and 12 eyes in the GWI group (control group).  18 patients (78.3%) were male and 5 (21.7%) were female. The patients’ ages ranged from 40 to 77 years (55.45 ± 9.5). 10 (43.5%) were unemployed and 3 (56.5%) were doing freelance. 9 (39.1%) had no education, 8 (34.8%) had primary school education, 2 (8.7%) had junior high school education and 4 (17.4%) had senior high school education.  Almost all patients had MB-type leprosy (21 patients or about 91.3%), and most of them had been diagnosed with leprosy for more than 5 years.  The inclusion  criteria were patients with paucibacillary (PB)- or multibacillary (MB)-type leprosy with unilateral/bilateral lagophthalmos who had not undergone eyelid reconstruction, patients aged 18 years or older, and who could undergo surgery with local anesthesia. | Modified tarsorrhaphy versus gold weight implant technique. | Lagophthalmos distance decreased in the MT (3.09 mm to 0.43 mm) and GWI groups (3.21 mm to 0.83 mm) at postoperative year 1.  The MT and GWI techniques showed no significant difference in decreasing lagophthalmos distances with or without gentle pressure at nasal, central, and temporal areas.  Ocular Surface Disease Index score, tear break-up time, and Schirmer test without and with anaesthesia in the MT and GWI groups showed a *p*-value of > 0.05.  OSDI score showed significant improvement from pre- to post-surgery within each group, with no significant difference between them.  Epitheliopathy improvement occurred in 54.55% of MT group and 58.33% of GWI group. Corneal sensitivity change in the inferior quadrant of the MT group (50.00 to 51.30 mm) and in the GWI group (49.61 to 52.93 mm) resulted in a *p* > 0.05 | 1 year | No complication found in the MT group, while two eyes (15%) in the GWI group experienced implant extrusion.  Patients in GWI group experienced limited vision field when the eyes were open due to the weight in their upper lids.  Tear film stability was less than normal, in both groups at preoperative assessment, but this increased insignificantly post-surgery. | The MT technique is as effective as the GWI technique but more efficient than the GWI technique as a surgical treatment for paralytic lagophthalmos in patients with leprosy.  Surgery duration of both techniques was similar and surgery cost in the MT and GWI groups yielded a *p* < 0.05.  Cost was significantly higher in the GWI group since gold as the implant material, which is a higher value, was used in the GWI surgery, even though the surgery preparation cost (laboratory examinations, radiology imaging) of both MT and GWI procedures were similar. Moreover, for two patients who experienced implant extrusion and underwent an additional MT procedure, the total cost of surgery in the GWI group was higher.  Final mean cost of those in the GWI group after complication correction was significantly higher (3.017.437,54 ± 560.823,97 IDR) than of those in the MT group (p < 0.05).  A patient belonging to the GWI group had discomfort at the 3-month evaluation with very limited vision field, which interfered with his job as a motorbike driver, and requested to receive the MT surgery. Surgery was performed and the patient felt more comfortable after the second surgery.  Common complication in the GWI group was allergic reactions. Therefore, an allergy examination is necessary.  MT technique is recommended as an alternative treatment for paralytic lagophthalmos in patients with leprosy as this technique was as effective as the GWI technique but more efficient than the GWI technique. The MT group showed no complication and felt more satisfied with the results than those in the GWI group. |
| Gold weight implants in the management of lagophthalmos in leprosy patients  Essam El Toukhy  [10] | Prospective study of 12 leprosy patients with gold weight implants | Cairo, Egypt  Outpatient procedures in the leprosy clinic, Kalaa, Cairo | 12 patients with leprosy and lagophthalmos over 5mm. All reside in a leprosarium, and all finished their MDT therapy 5–10 years ago. All males, aged from 38–73 years (mean 46 years). Seven patients had been treated as paucibacillary (PB) and five as multibacillary (MB) leprosy. | Gold weight implants | At 3 months follow-up, 11 had satisfactory closure (defined as a reduction of the lid gap of 3 mm or more). Complete lid closure was achieved in eight out of the 12 cases; this resulted in improvement of corneal irritation and good eye coverage particularly during sleep. Incomplete closure was attained in four patients, however, they all had similar improvement in the corneal manifestations. | 1 year | Six out of 12 implants were extruded within the first year. Two more implants had to be removed due to chronic inflammatory reaction. This lowered the success rate to 33% (4/12 cases) after 1 year.  The high incidence of extrusion and inflammatory reactions that occurred may be related to poor socio-economic state and the high incidence of infections occurring in them.  Some minor transient complications in the form of postoperative lid oedema and/or ecchymosis which resolved spontaneously. | Lateral tarsorrhaphy is simple but produces a poor cosmetic outcome. Horizontal lower lid shortening is more complex but works only on the lower lid and cannot correct lagophthalmos more than 3 mm. Temporalis transfer is more complex and requires a long period of intensive physiotherapy.  The Gold weights implants procedure is simple, effective, is done under local anesthesia and can be used with other lower lid procedures.  In our study, gold implantation had a low success rate. Careful selection of cases to decrease incidence of infection and/or extrusion is needed. |
| Surgical treatment of bilateral paralytic lagophthalmos using scapha graft in a case of lepromatous leprosy  Laura Lavilla 1 , Jesús Castillo, Angel M Domínguez, Nelson A Rodríguez, Francisco J Ascaso, José A Cristóbal  [11] | Case Report | Spain,  Department of Ophthalmology, ‘Lozano Blesa’ University Clinic Hospital, Zaragoza | 36-year-old Mozambican man | Surgical correction of paralytic lagophthalmos with a graft obtained from the scapha of the ear. | The symptoms disappeared because a good protection of the eyeball was achieved. The donor defect healed satisfactorily without deforming the ear. At 3-year follow-up, the results thus far appear long lasting. | 3-year follow-up | No postoperative complications. | Leprosy patients with paralytic lagophthalmos refractory to conventional repair can be treated using an auricular graft from the scapha with good aesthetic and functional results.  We would recommend the simple resection of retractors technique, for an improvement of 1 to 2 mm of scleral show. When improvements greater than 2mm are necessary we prefer the more complete operation, posterior lamellar spacer graft and lateral eyelid tightening.  Graft from the auricle is easily harvested and provides an excellent and simple method for repairing lower eyelid retraction. It does not contract and has minimal donor site morbidity. |
| Reanimation of the lagophthalmos using stainless steel weight implantation; a new approach and prospective evaluation  Seree Kuntheset  [12] | Prospective study | Thailand,  Phra-Pradaeng Hospital between January 1993 and February 1998 | 20 Thai patients. 18 leprosy patients and two patients without leprosy (four females and 16 males). Only one eye was operated on in each of 18 patients and both eyes in two patients were operated on for a total of 22 eyes. The patients ranged in age from 29 to 65. | Stainless steel weight implantation | The surgery was graded as excellent if complete closure was obtained, moderate if a residual eyelid palpebral fissure distance of 0.5-1.5 mm persisted. Both excellent and moderate results are accepted as a success, and the success rate is, therefore, about 90% in this study. After more than 12 months of follow up, 20 of the 22 eyes were still in good condition. In cases with excellent results, some of the patients with previously keratinized corneas were noted to have the keratinization disappear. | 7 to 55 months | In two eyes after 7-8 months, the plates were removed due to thinning of the skin and tightening of the lid.  Post-operative complications, such as ectropion, entropion and overtightening, were not found in this study. Finally, if there are some post-operative problems, removal of the plate is simple, and the weight can be increased or decreased easily on follow-up surgery. | This study showed that a stainless-steel plate is an alternative to gold for the weight implant technique.  The advantage of this surgical technique is that it is a relatively straightforward technique that is familiar to ophthalmic surgeons. Readjustment of the prosthesis usually is not necessary once the weight and technique are optimal. This procedure does not require physical therapy for success and has a good cosmetic effect.  The disadvantages include the possibility of infection and extrusion. However, this has represented a potential rather than a real problem in both this study and other studies to date. There is also some concern about cosmetic appearance. If the weight implant is placed too superficially, it may resemble a chalazion-like subcutaneous lump in the eyelid. The patient may have an ongoing need for topical lubricant drops and ointments. Some patients have developed increased and variable astigmatic refractive error related to the gravitational effect of the weight implant over the corneal surface curvature.  There were no signs of inflammation in any of the 22 eyes during the period of follow up. Pure gold (medical grade) is expensive, and there is a problem in availability, but it is the best material if the patient can afford it. Stainless steel is the alternative choice because it is inexpensive, readily available, and produces a favourable result. |

References

1. Andersen JG. Surgical treatment of lagophthalmos in leprosy by the Gillies temporalis transfer. Br J Plast Surg. 1961;14: 339–345. doi:10.1016/s0007-1226(61)80057-x

2. Das P, Kumar J, Karthikeyan G, Rao PSS. Efficacy of temporalis muscle transfer for correction of lagophthalmos in leprosy. Lepr Rev. 2011;82: 279–285.

3. Singhal M, Bhattacharya R, Jain S. Dynamic temporalis muscle transfer revisited – a Technique for correction of lagopthalmos in leprosy patients. Int J Recent Trends Sci Technol. 2015;14: 344–347.

4. Gupta RC, Kushwaha RN, Budhiraja I, Gupta P, Singh P. Modified silicone sling assisted temporalis muscle transfer in the management of lagophthalmos. Indian J Ophthalmol. 2014;62: 176–179. doi:10.4103/0301-4738.128629

5. Verma N, Garg SP, Kalra VK, Fromberg G. An evaluation of Gillies’ procedure for lagophthalmos in leprosy. Indian J Ophthalmol. 1984;32: 368–370.

6. Ahn SY, Park HJ, Kim JP, Park TH. Temporalis Muscle Transfer for the Treatment of Lagophthalmos in Patients With Leprosy: Refinement in Surgical Techniques to Prevent Postoperative Ptosis. J Craniofac Surg. 2016;27: 94–96. doi:10.1097/SCS.0000000000002271

7. Jue M-S, Yoo J, Kim M-S, Park H-J. The Lateral Tarsal Strip for Paralytic Ectropion in Patients with Leprosy. Ann Dermatol. 2017;29: 742–746. doi:10.5021/ad.2017.29.6.742

8. Soares D, Chew M. Temporalis muscle transfer in the correction of lagophthalmos due to leprosy. Lepr Rev. 1997;68: 38–42. doi:10.5935/0305-7518.19970006

9. Irawati Y, Natalia MER, Gondhowiardjo TD, Dachlan I, Soebono H. Modified tarsorrhaphy versus gold weight implant technique for paralytic lagophthalmos treatment in patients with leprosy: One-year observation of a randomized controlled trial study. Front Med. 2022;9: 941082. doi:10.3389/fmed.2022.941082

10. El Toukhy E. Gold weight implants in the management of lagophthalmos in leprosy patients. Lepr Rev. 2010;81: 79–81.

11. Lavilla L, Castillo J, Domínguez AM, Rodríguez NA, Ascaso FJ, Cristóbal JA. Surgical treatment of bilateral paralytic lagophthalmos using scapha graft in a case of lepromatous leprosy. Lepr Rev. 2009;80: 448–452.

12. Kuntheseth S. Reanimation of the lagophthalmos using stainless steel weight implantation; a new approach and prospective evaluation. Int J Lepr Mycobact Dis Off Organ Int Lepr Assoc. 1999;67: 129–32.
