# Supplementary material for: Techniques for the surgical correction of lagophthalmos secondary to leprosy: A systematic review
Source: PLoS Negl Trop Dis. 2025 Jun 16;19(6):e0013200. doi: 10.1371/journal.pntd.0013200 (PMC12193040; doi:10.1371/journal.pntd.0013200)
Supplement: Attachment — Submitted filename: Response to Reviewers.docx [file pntd.0013200.s006.docx]

The Editor, Reviewers,

PLoS Neglected Tropical Diseases,

Date: April 16th, 2025

Subject: Addressing Reviewer Comments for PNTD-D-25-00162

Dear Editor, Reviewers,

Many thanks for the feedback provided on the manuscript, we feel it has been extremely constructive, and the changes suggested have helped improve the manuscript as a whole. We address each of the comments point by point below and would be open to receiving more feedback on the changes we have implemented.

Kind regards,

Authors of PNTD-D-25-00162

**Reviewer’s original comments are in black with the authors’ response in red below each point raised.**

**Methods**

Reviewer #1: Study design is appropriate, but available literatures are limited
Most are retrospective study. One case report Scapha grafting may not appropriate to include in this revision.

We thank reviewer 1 for their constructive comments. Regarding the paper on Scapha grafting it meets our inclusion criteria and since the paper meets our criteria, we feel it is important to include. We made sure to separate it into “other” techniques to provide separation from more commonly performed procedures.

Reviewer #2: • What experiments or interventions were used? NA
• Are the experiments or interventions appropriate for addressing the research question? NA
• Are conditions adequate and the right controls in place? NA
• Is there enough data to draw a conclusion? 12 articles were selected; in this review this seems adequate. Perhaps French literature would have provided extra info?
• Do the authors address any possible limitations of the research? NO, but not needed
• Was data collected and interpreted accurately? Yes, in a very accurate way!
• Do the authors follow best practices for reporting? Yes
• Does the study conform to ethical guidelines? NA
• Could another researcher reproduce the study with the same methods?
In other words, have the authors provided enough information to validate
the study? YES

We thank the reviewer for their constructive feedback. We agree including foreign language papers would potentially return more papers and as such have included this as a limitation of the study.

Reviewer #3: The objectives are clearly written, study design is also well written.
Based on prisma checklist, did you use any method to assess certainty (or confidence) in the body of evidence for an outcome?

We thank reviewer 3 for their constructive feedback. We did not conduct a grade assessment as we did not feel the body of evidence was big enough to base any solid recommendations on the review. Hence, in our recommendations we emphasise the need for clinicians to best assess the value of each technique within their own circumstances and population. GRADE is not an essential component under the PRISMA checklist, and we have as such marked it as “Not completed” in the checklist attached.

**Results**

Reviewer #1: Although enough, but Technique of operations and complication in table or image will be helpful for better understanding

We have added an additional table, Table 1, to the results section. Additionally, all requested information can already be found in S3.

Reviewer #2: • Do the results support the conclusions? YES
• Do the conclusions overreach? NO
• Do the authors discuss any limitations of the study? YES
• If the journal selects based on advance in the field does the study demonstrate this advance? IT IS A GOOD REVIEW, ANY ADVANCE IS NOT DISCUSSED.

We thank the reviewer for their comments.

Reviewer #3: The analysis presented match the analysis plan and clearly presented. However maybe It might be helpful to include a brief explanation regarding potential differences in GWI usage across cases or groups. Additionally, consider discussing whether there is any observed correlation between GWI usage and inflammation, particularly in relation to the possibility of extrusion due to foreign body response.
Also for the table, I think it would be good to add extra rows for success rate for each methods, patient characteristics, complications for each study.

We thank the reviewer for their comments. We have added an additional table to the results section to make it easier for the reader to interpret the results. Given the small sample size and difficulty in comparing the groups we did not comment on any potential correlation as we did not feel this was possible from the included data.

**Conclusions**

Reviewer #1: As there were limited number of publications, conclusion drawn from review is clear.

We thank the reviewer for their comments.

Reviewer #2: (No Response)

Reviewer #3: The conclusion is well supported by data presented. Are there any more limitations on this study? Please elaborate more on why meta-analysis couldn’t be performed. Also highlight on how choosing the most appropriate surgery technique is important to achieve the best result functionally and aesthetically

We could not compare like with like due to the varying reporting methods used by the included studies, additionally for side effects multiple studies did not report incidence. We have thus expanded on the limitations as follows: “Due to the nature of the data presented and the varying outcome measures in the included studies it was also not possible to perform a meta-analysis of the pooled results.”

We have additionally added a sentence to the discussion: “Within the techniques a range of aesthetic and functional outcomes are noted. Where patients are able to be followed up and resources allow, more resource intense but functionally and aesthetically preferrable procedures such as TMT may be preferred.”

**Editorial and Data Presentation Modifications?**

Reviewer #1: If there will be table regarding Technique of operation, retrospective/prospective, number of cases, complications, outcome, may be more helpful for readers

We have added a table to the results section. This information is additionally already present as S3 but we have summarised this to a shorter table that is now presented in the results section.

Reviewer #2: (No Response)

Reviewer #3: Minor revision

**Summary and General Comments**

Reviewer #1: Overall, it is a good systematic review based on available publications. Minor revision is needed.

We thank the reviewer for their comments.

Reviewer #2: Some personal notes:

When faced with lagophthalmos ( in general caused by leprosy neuritis) we start with an analysis of the degree of the lid gap when the eyes are gently closed and with maximal strength, and signs of corneal damage due to dryness/infection.

When the lid gap is max 3 mm and correction is indicated I would recommend a simple medial canthoplast.
When the gap is 3-6 mm, in general a lateral suspension canthoplasty perhaps in combination with a medial suspension canthoplasty is performed. In this a suture Nylon 5x0 is fixed to the periosteum and woven through the lower orbicularis muscle and back again and knotted. When doing only a lateral suspension one should not put any lateral stretch to the entry of the lacrimal duct.
When the lidgap is >6mm, a TMT is indicated.

You make a very valid point that too often there is little expertise and when local colleagues want to be trained one may want to create a routine in learning a not too complex technique. For this reason we often opt for a simple canthoplasty or suspension plasties as these are easier to master, do not ask for post-op physiotherapy, easier follow up, and are thus more affordable that TMT’s (or facial sling procedures). Another important limitation is often poverty and therefore no money for transport, and the absence of a health insurance which makes day care mandatory.
In this it is after the operation accepted that often a remaining lid gap of a 2? mm is accepted as long as the cornea is well covered and protected.

We thank the reviewer for their detailed comments and personal insights. We feel the discussion already addresses most of the points raised and hope that the paper can serve as a point of discussion in the literature to promote further research to address the points raised above.

Reviewer #3: Overall, the paper is well written

We thank the reviewer for their comments.
